# The Dimerization Domain in DapE Enzymes Is required for Catalysis

**DOI:** 10.1371/journal.pone.0093593

**Published:** 2014-05-07

**Authors:** Boguslaw Nocek, Anna Starus, Magdalena Makowska-Grzyska, Blanca Gutierrez, Stephen Sanchez, Robert Jedrzejczak, Jamey C. Mack, Kenneth W. Olsen, Andrzej Joachimiak, Richard C. Holz

**Affiliations:** 1 Center for Structural Genomics of Infectious Diseases, Computation Institute, University of Chicago, Chicago, Illinois, United States of America; 2 The Department of Chemistry and Biochemistry, Loyola University-Chicago, Chicago, Illinois, United States of America; 3 The Midwest Center for Structural Genomics, Bioscience Division, Argonne National Laboratory, Lemont, Illinois, United States of America; 4 Department of Chemistry, Marquette University, Milwaukee, Wisconsin, United States of America; Rochester Institute of Technology, United States of America

## Abstract

The emergence of antibiotic-resistant bacterial strains underscores the importance of identifying new drug targets and developing new antimicrobial compounds. Lysine and *meso*-diaminopimelic acid are essential for protein production and bacterial peptidoglycan cell wall remodeling and are synthesized in bacteria by enzymes encoded within *dap* operon. Therefore *dap* enzymes may serve as excellent targets for developing a new class of antimicrobial agents. The *dapE*-encoded *N*-succinyl-L,L-diaminopimelic acid desuccinylase (DapE) converts *N*-succinyl-L,L-diaminopimelic acid to L,L-diaminopimelic acid and succinate. The enzyme is composed of catalytic and dimerization domains, and belongs to the M20 peptidase family. To understand the specific role of each domain of the enzyme we engineered dimerization domain deletion mutants of DapEs from *Haemophilus influenzae* and *Vibrio cholerae*, and characterized these proteins structurally and biochemically. No activity was observed for all deletion mutants. Structural comparisons of wild-type, inactive monomeric DapE enzymes with other M20 peptidases suggest that the dimerization domain is essential for DapE enzymatic activity. Structural analysis and molecular dynamics simulations indicate that removal of the dimerization domain increased the flexibility of a conserved active site loop that may provide critical interactions with the substrate.

## Introduction

Emerging antibiotic resistance has been recognized as a world-wide health issue since the introduction of penicillin more than 80 years ago [1], [2]. Most importantly, the rapid emergence of resistant bacteria makes today's antibiotics more and more ineffective, consequently increasing the need for a novel class of antibacterial agents [3]. Currently available antibiotics target only a limited number of microbial pathways and employ two major strategies: (i) inhibition of cell wall remodeling and (ii) inhibition of protein synthesis. As a result, only two new classes of antibacterial drugs have reached the market since 1962. The Center for Disease Control and Prevention (CDC) recently reported the emergence of several strains of *Staphylococcus aureus* that are resistant to even the most powerful antibiotic of last resort, vancomycin. These cases emphasize the fact that no drug can prevent a simple staph infection from becoming deadly [4]. According to the Infectious Diseases Society of America, a minimum of ten new systemic antibacterial drugs need to enter the market by the year 2020 in order to maintain proper control of infectious diseases. Therefore, development of new classes of inhibitors that target essential metabolic pathways and unique enzymes is critical in order to maintain control of infectious diseases [5], [6].

The lysine biosynthetic pathway offers several enzymes that could serve as potential drug targets [7], [8]. Two products of this pathway, lysine and *meso*-diaminopimelate (*m*DAP), are essential for protein and peptidoglycan cell wall synthesis in Gram-negative and most Gram-positive bacteria. Many bacteria, plants and algae synthesize lysine and *meso*-diaminopimelic acid (mDAP) from succinic acid [9], [10], [11]. In contrast, lysine is not synthesized in humans but it is an essential amino acid, therefore it must be ingested. It has been shown that deletion of the *dapE* gene in the mDAP/lysine biosynthetic pathway that encodes the *N*-succinyl-L,L-diaminopimelic acid desuccinylase (DapE) is lethal in *Helicobacter pylori* and *Mycobacterium smegmatis* [12], [13]. DapE hydrolyzes *N*-succinyl-L,L-diaminopimelic acid to L,L-diaminopimelic acid and succinate, is part of a biosynthetic pathway that is the major source of lysine in bacteria, and is essential for cell growth and proliferation. Since there are no similar biosynthetic pathways in mammals, inhibitors that target DapEs are hypothesized to exhibit selective toxicity against bacteria and have little or no effect on humans [9], [14]. DapE coding genes have been identified in all pathogenic Gram-negative bacteria, and the enzyme has been purified and characterized from several sources [8]. Of particular interest are DapEs from the “ESKAPE” pathogens (***E*** *nterococcus faecium*, ***S*** *taphylococcus aureus*, ***K*** *lebsiella pneumonia*, ***A*** *cinetobacter baumanii*, ***P*** *seudomonas aeruginosa* and ***E*** *nterobacter* species), which account for more than 60% of the antibiotic resistant hospital acquired infections in the United States (13–18). Alignment of the DapE gene from *Haemophilus influenzae* (*Hi*DapE) with the gene sequences of DapEs from “ESKAPE” pathogens reveals at least 49% identity [15].

All the DapE enzymes characterized to date belong to the M20 family of dinuclear Zn(II)-dependent metalloproteases. Structural studies of M20 metalloproteases showed that these enzymes exist as dimers comprised of catalytic and dimerization domains or monomers having a single catalytic domain (16). Structural studies on *Hi*DapE revealed that the enzyme belongs to the former group composed of a larger thioredoxin-like 3-layer (αβα) sandwich domain that carries out catalysis and a smaller ferrodoxin-like domain providing the dimer interface [16]. The core of the catalytic domain consists of an eight-stranded twisted β-sheet that is sandwiched between seven α-helices and houses the active site. The active site is constituted by residues from five loops, contains two zinc ions, and is exposed to the solvent.

The architecture of the active site of *Hi*DapE and the core of the catalytic domain are strikingly similar to the aminopeptidase from *Aeromonas proteolytica* (AAP) and the carboxypeptidase from *Pseudomonas sp* strain-RS-16 (CPG2) [17], [18], even though they catalyze markedly different hydrolytic reactions. One explanation for this similarity is the highly flexible design of their active sites, which are constructed by five loops. This design preserves the dinuclear catalytic core and at the same time allows for evolution of specificity for new substrates without the need for major modifications to the overall structure. The flexibility of the active site is especially relevant from the perspective of understanding substrate recognition and binding, which is directly related to inhibitor design. In order to examine the role of the catalytic domain of DapE enzymes and the role of the active site loops, we have engineered the *Hi*DapE and the DapE from *Vibrio cholerae* (*Vc*DapE) by deleting their dimerization domains. This truncation leads to protein constructs that have only the catalytic domain, are monomeric in solution, and closely resemble AAP. Kinetic characterization of the truncated DapEs (*Vc*DapE^T^ and *Hi*DapE^T^) shows that the catalytic domain alone is completely inactive. X-ray crystallographic data and molecular dynamic simulations along with site-directed mutagenesis studies provide insight into the role of the dimerization domain and underlying importance of the active site loops in catalysis.

## Materials and Methods

### Gene cloning and protein expression

The coding genes of full-length *Vc*DapE and *Hi*DapE were cloned into vector pMCSG7 and amplified by PCR from *V. cholerae* O1 biovar El Tor str. N16961 (ATCC) and from *H. influenzae strain (ATCC)*, respectively, with *KOD* DNA polymerase using conditions and reagents according to the standard protocol described previously [19]. The truncated version of *Hi*DapE^T^ (residues 181–294 deleted) and *Vc*DapE^T^ (residues 181–295 deleted) were designed based on the full-length *Hi*DapE structure and were cloned using the same procedure. These deletion proteins were reengineered in such a way that the C-terminal domain was truncated at the connector region between the dimerization and the catalytic domains and replaced with a GG linker. The deletion of the region coding for the dimerization domain was achieved *via* PCR of pMCSG7-*Vc*DapE and pMCSG7-*Hi*DapE according to the previously described protocol [20]. The following primers were used: *Vc*DapE^T^ 181–295; 5′TGGCGGTGGCTTCCTGACCGATACGGGCGA3′ and 5′GGAAGCCACCGCCACGACGGCCATTTTTCACC3′, *Hi*DapE^T^ 181–294; 5′CGGCGG TGGCTTTTTAACAAAACCAGGTAAATTATTAGATTCGATAACC3′ and 5′AAAAAGCCACC GCCGCGGCGACCATTTTTGAC3′. This process generated expression clones of fusion proteins that have an N-terminal His_6_-tag, a TEV protease recognition site (ENLYFQ↓S), followed by the DapE catalytic domain. All proteins were expressed in an *E. coli* BL21(DE3)-derivative that harbors the pMAGIC plasmid encoding one rare *E. coli* Arg tRNA (covering codons AGG/AGA). The transformed BL21(DE3) cells were grown at 37°C in Luria-Bertani medium and protein expression was induced with 1 mM isopropyl-β-d-thiogalactoside (IPTG). The cells were then incubated and shaken vigorously at 18°C overnight. The harvested cells were re-suspended in lysis buffer (500 mM NaCl, 5% (v/v) glycerol, 50 mM HEPES, pH 8.0, 10 mM imidazole, 10 mM 2-mercaptoethanol) and stored at −80°C.

### Purification of *Vc*DapE, *Vc*DapE^T^ and *Hi*DapE^T^

The *Vc*DapE, *Vc*DapE^T^ and *Hi*DapE^T^ proteins were purified according to the standard protocol for Ni-NTA affinity chromatography, as described previously [19]. The His_6_-tag was removed by treating each enzyme with His_6_-tagged TEV protease for 16 h at 4°C in 50 mM HEPES, pH 8.0. Cleaved protein was separated from TEV using Ni-NTA affinity chromatography. After Ni-NTA column chromatography, *Vc*DapE^T^ and *Hi*DapE^T^ were further purified by size-exclusion chromatography on a HiLoad 16/600 Superdex 200 Prep Grade (GE Healthcare) using standard crystallization buffer (250 mM NaCl, 20 mM HEPES pH 8.0, and 1 mM TCEP) and concentrated to 25 mg/ml. The full-length *Vc*DapE protein was purified according to the same procedure however the size-exclusion chromatography step revealed formation of “soluble aggregates”. Therefore, the buffer conditions were altered such that the salt concentration was increased to 800 mM NaCl and glycerol (10%) was added, allowing for purification of small quantities of soluble protein.

### Kinetic characterization of *Vc*DapE, *Vc*DapE^T^, and *Hi*DapE^T^

Recombinant WT-*Vc*DapE, *Vc*DapE^T^, and *Hi*DapE^T^ were characterized biochemically by monitoring amide bond cleavage of L,L-SDAP at 225 nm (ε = 698 M^−1^ cm^−1^). The assay was performed in triplicate in 50 mM phosphate buffer, pH 7.5, in the presence of two equivalents of Zn(II). Enzyme activities were expressed as units/mg where one unit is defined as the amount of enzyme that releases 1 µmol of L,L-SDAP at 25°C in 1 min. *K*_m_ and *k*_cat_ values were obtained by fitting initial rates data to the Michaelis-Menten equation using Origin software [21], [22], [23]. Catalytic activities were determined with an error of ±10%.

### Protein crystallization

The sitting-drop vapor-diffusion method was used to obtain crystals of *Vc*DapE^T^ and *Hi*DapE^T^ at 16°C using a Mosquito liquid handling robot with 96 well plates. Crystals of both the apo- and dinuclear Zn(II)-loaded forms of *Vc*DapE^T^ were obtained using 400 nl of a precipitant solution (20% (v/v) 1,4-butanediol, 0.1 M sodium acetate pH 4.5) and 400 nl a 19 mg/ml solution of *Vc*DapE^T^ in crystallization buffer, within 14 days. In order to grow the crystals of dinuclear Zn(II)-loaded forms of *Vc*DapE^T^, a 1 mM ZnCl_2_ solution was added to the protein solution.

Crystals of *Hi*DapE^T^ were obtained using 400 nl of a precipitant solution (0.2 M ammonium acetate, 0.1 M BIS-TRIS pH 5.5, 25% (w/v) polyethylene glycol 3350) and 400 nl of a 15 mg/ml solution of *Hi*DapE^T^ within two weeks. The asymmetric unit contains two monomeric molecules of the *Hi*DapE^T^. For cryo-protection, all crystals were transferred to the mother liquid containing a 25% mixture of glycerol and ethylene glycol.

### Data collection and structure determination

Prior to data collection, the X-ray florescence spectrum was recorded for *Vc*DapE^T^, *Hi*DapE^T^ and apo-*Vc*DapE^T^ crystals, which identified the presence of Zn ions in the protein crystals of *Vc*DapE^T^ and *Hi*DapE^T^. Data collection was carried out on the 19-ID beam line of the Structural Biology Center at the Advanced Photon Source according to procedures described previously [16]. Data were collected at a wavelength of 0.98 Å from the single crystals and were processed using HKL3000. Crystallographic parameters are summarized in Table 1. Initially, data for *Vc*DapE^T^ and *Hi*DapE^T^ were processed, scaled and the structures refined in the space group P3_2_12. Even though these models refined with low R-factors (R_cryst_/R_free_ = ∼16/18%), spurious density features were observed in the electron density maps. Re-examination of these data suggested a twinning test be performed using Xtriage in Phenix [24], which indicated twinning. The twinning operator is parallel to the two-fold axis making the space group pseudo P3_2_12. Therefore, data were rescaled in the space group P3_2_. The structures of *Vc*DapE^T^ and *Hi*DapE^T^ were determined by molecular replacement using the catalytic domain of *Hi*DapE (PDB ID 3IC1) as a search model [16]. Molecular replacement searches were completed using MOLREP of the CCP4 suite [25], [26]. The initial models were rebuilt manually and refined using programs REFMAC 5.5 [27] and Phenix [24]. The final models were refined against all reflections except for 5% randomly selected reflections, which were used for monitoring *R*_free_. The final rounds of refinement were carried out using TLS refinement with 5 TLS groups. The final refinement statistics for all structures are presented in Table 1. Analysis and validation of the structures were performed with the aid of MOLPROBITY and COOT validation tools. Figures were prepared using Pymol.

**Table 1:** Data and Refinement Statistics.

Data collection statistics	*Vc*DapE^T^-apo	*Vc*DapE^T^-ZnZn	*Hi*DapE^T^-ZnZn
Space group	P3_2_	P3_2_	P2_1_
Unit cell (Å)	a = 49.6	a = 49.9	a = 61.7
	b = 49.6	b = 49.9	b = 44.7
	c = 232.6	c = 231.8	c = 92.5
			β = 92.9°
Resolution (Å)	37.6-1.65	40-1.65	30-1.84
Wavelength (Å)	0.98 Å	0.98	0.98
Number of observed reflections	523779	299329	99336
Number of unique reflections	77006	70716	42453
Redundancy^b^	6.8 (3.8)	4.2(1.8)	2.3(2.2)
*R*_merge_ a ^,^ b (%)	5.9 (50.1)	5.1 (26.0)	9.2 (55.7)
*R_rim_* a ^,^ b (%)	6.4 (56.7)	5.8 (35.5)	10.5(82.6)
*R_pim_* a ^,^ b (%)	2.4 (25.8)	2.6 (23.1)	6.5(51.0)
Completeness^b^ (%)	99.6 (93.8)	91.3 (49.2)	96.5 (97.8)
*I*/σ	40 (2.3)	23.2 (2.1)	9.5 (2.1)
**Phasing**			
phasing method	**MR**	**MR**	**MR**
**Refinement statistics**			
*R*_cryst_ (%)	13.81	14.32	19.7
*R*_free_ (%)	17.13	16.59	24.9
protein residues	532	513	505
zinc ion/acetate/butanediol/glycerol/ethanediol/	-/1/6/2/4	2/-/6/3/3	4/-/-/-/-
solvent	541	541	284
**Rmsd from target values**			
bond lengths (Å)	0.020	0.020	0.021
bond angles (deg)	2.11	1.43	2.03
**Average ** ***B*** ** factors (Å^2^)**			
protein	14.78	14.77	27.7
Zn	-	13.93	26.1
H_2_O	28.10	24.19	38.7
**PDB ID**	4ONW	4OP4	4H2K
Ramachandran (%)^c^ M.F./A.A.	97.5/2.5	97.3/2.7	96.8/3.0

a *R*_merge_ = Σ*_hkl_*Σ*_i_*|*Ii*_(_ *hkl*_)_−〈*I_hkl_*〉|/Σ*_hkl_*Σ*_i_I_i_*_(*hkl*)_, where I*i*_(_ *hkl*_)_ is the *i*th observation of reflection *hkl*, and 〈*I_hkl_*〉 is the weighted average intensity for all observations *i* of reflection *hkl*.

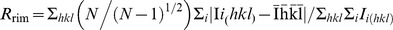
, and 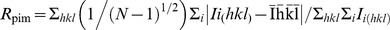.

b Numbers in parentheses are values for the highest-resolution bin.

c As defined by MOLPROBITY (M.F. –the most favored/A.A additionally allowed).

### Protein Data Bank accession code

The atomic coordinates and structure factor file for the structure of the catalytic domain of apo-*Vc*DapE^T^, ZnZn-*Vc*DapE^T^ and ZnZn-*Hi*DapE^T^ have been deposited in the RCSB Protein Bank with accession code 4ONW, 4OP4 and 4H2K, respectively.

### Determination of Molecular Weight of *Vc*DapE, *Vc*DapE^T^ and *Hi*DapE^T^

The molecular weight (MW) of the *Hi*DapE, *Hi*DapE^T^ and *Vc*DapE^T^ proteins in solution were determined using size-exclusion chromatography (SEC) on a HiLoad 16/600 Superdex 200 Prep Grade column (GE Healthcare) in the crystallization buffer at 5 mg/ml concentrations. The column was calibrated with aprotinin (6.5 kDa), ribonuclease A (13.7 kDa), carbonic anhydrase (29 kDa) ovalbumin (43 kDa), conalbumin (75 kDa), aldolase (158 kDa), ferritin (440 kDa), and thyroglobulin (669 kDa) as standards. A calibration curve of *K_av_ vs.* log MW was prepared using the equation *K*_av_ = *V*_e_−*V*_o_/*V_t_*−*V*_o_, where *V*_e_ = elution volume for the protein, *V*_o_ = column void volume, and *V*_t_ = total bed volume (Supplementary Materials Figure S1A in File S1).

### Dynamic light scattering (DLS) assessments

DLS analyses were performed using the DynaPro-801 molecular sizing instrument (Protein Solutions) at 21°C. Aliquots of protein samples collected from the SEC column were concentrated to a final concentration of 2.0 mg/ml in the crystallization buffer. The solutions were filtered with Ultrafree-MC microcentrifuge filters (0.22 µm, Millipore) and centrifuged at 20,000 *g* for 10 min at 4°C and before measurements were taken. Hydrodynamic radii (*R_H_*), degree of sample polydispersity and MWs were calculated using the manufacturer's software version 5.25.44.

### Molecular modeling and dynamics simulations

The structure of the catalytic domain of *Vc*DapE^T^ is well ordered and excellent electron density is observed for protein main-chain, side-chains, metal, phosphate ions, and water molecules. Using the VMD molecular graphics program [28], the protein model with its hydrogen atoms was created and placed in a water-box containing 7826 TIP3 water molecules [29], 13 sodium, and 2 chloride ions to neutralize the charge and provide counter ions. The charges on the histidine residues were determined by inspection of their local environments. The energy of this initial structure was minimized with 3,000 steps of conjugated gradient minimization using the CHARMM27 force field [30] and the NAMD molecular dynamics (MD) program [31]. The structure was relaxed by gradually heating it from 10 to 310 K in steps of 10 K with 1,000 steps of molecular dynamics at each temperature. Periodic boundary conditions were used. The cutoffs for non-bonding (van der Waals and electrostatic) interactions were 12 Å. The switch distance was 10 and 1.0 Å using a 1–4 scaling factor. The time step was 2 fs. These conditions were used for all of the molecular dynamics simulations. After 10,000 steps of pressure and temperature equilibration using a Langevin piston, the system was subjected to 30,000 steps of molecular dynamics. This process constitutes equilibration of the structure. The structures for *Hi*DapE^T^ and *Hi*DapE were also equilibrated in the same manner. Since these X-ray structure models had a few loops missing due to the poor electron density maps, the missing regions were modeled using the modeling tools found in Swiss PDB Viewer [32]. The complete *Vc*DapE structure was produced through homology modeling, using *Hi*DapE as a template through the automated mode of the Swiss Model service [33]. The two chains of *Vc*DapE were produced separately from each other, combined using VMD, and equilibrated in the manner previously described. The X-ray structure for AAP (PDB ID 1RTQ) [18] was used as a standard for comparison as AAP and dinuclear Zn(II)-loaded *Vc*DapE^T^ and *Hi*DapE^T^ are characterized by similar active sites and sequence lengths.

For each of these five structures, 5 ns of simulation data were accumulated. The rmsd per residue values were calculated for MD production runs of the four structures using the VMD molecular graphics program [28]. These calculations were used to determine structural changes between each of the five structures. MOLMOL plots [34] were also generated for each structure to give a visual depiction of the structural changes, especially for areas of large movement. For these diagrams 171 equally spaced snapshots over a 5 ns simulation were used for each of the five proteins. Three of the simulations (ZnZn(*Vc*DapE^T^), *Hi*DapE^T^ and *Hi*DapE) were run for 10 ns, but there were no significant differences in the flexibilities of these when the second 5 ns simulations were compared with the first 5 ns.

### Loop V Site Directed Mutagenesis

The site-directed mutants T325A, T325S and T325C of WT-*Hi*DapE were prepared using the Quick Change Site-Directed Mutagenesis Kit (Stratagene) following the procedure outlined by Stratagene using the following primers, T325A: 5′-ACAGGTGGCGGCGCGTCAGACGGTC-3′, 5′-GACCGTCTGACGCGCCGCCACCTGT-3′, T325S: 5′-ACAGGTGGCGGCTCGTCAGACGGTC-3′, 5′-GACCGTCTGACGAGCCGCCACCTGT-3′, T325C: 5′- GCTGAAACAGGTGGCGGCTGCTCAGACGGTCGTTTTATT-3′, 5′-AATAAAACGACCGTCTGAGCAGCCGCCACCTGTTTCAGC-3′. Polymerase Chain Reaction (PCR) was performed using 70 ng of a dsDNA template. PCR was performed at 95°C for 30 seconds followed by 16 cycles of (95°C for 30 seconds, 55°C for 1 minute, 68°C for 5 minutes) and final extension at 68°C for 6 minutes. Reaction products were transformed into BL21(DE3)/magic *E. coli* competent cells (Invitrogen) and grown on Luria-Bertani agarose plates containing ampicillin (100 µg mL^−1^) and kanamycin (25 µg mL^−1^). A single colony of each mutant was grown in 5 mL Luria-Bertani medium containing ampicillin (100 µg mL^−1^) and kanamycin (25 µg mL^−1^). Plasmids were isolated using the QIAprep-Spin Miniprep Kit (QIAGEN). The plasmid DNA was sent to the DNA sequencing facility (University of Chicago Cancer Research Center DNA Sequencing Facility, Chicago IL) to confirm the mutations. Glycerol stocks were prepared and stored at −80°C until further needed. Purification of *Hi*DapE T325A, T325C and T325S mutant enzymes and kinetic characterization was conducted as described above. Circular dichroism (CD) spectra were recorded on an Olis DSM-20 CD spectrophotometer at 25°C in 50 mM HEPES, 300 mM NaCl at pH 7.5 (Supplementary Materials Figure S1B in File S1).

## Results

### Kinetic characterization of wild-type and truncated DapE

Kinetic data were obtained for recombinant *Vc*DapE, *Hi*DapE, *Vc*DapE^T^, and *Hi*DapE^T^ by monitoring amide bond cleavage of L,L-SDAP at 225 nm. The *k*_cat_ value obtained for WT-*Vc*DapE was 80±10 s^−1^, with a corresponding *K_m_* value of 1.2±0.2 mM while the *k*_cat_ value obtained for *Hi*DapE was 114±10 s^−1^, with a *K_m_* value of 0.8±0.1 mM, which is in good agreement with previous studies [23] (Surprisingly, no detectable activity was observed for *Hi*DapE^T^ or *Vc*DapE^T^ under the standard assay conditions.

### Structure of the catalytic domain of *Hi*DapE^T^

**Figure 1:**
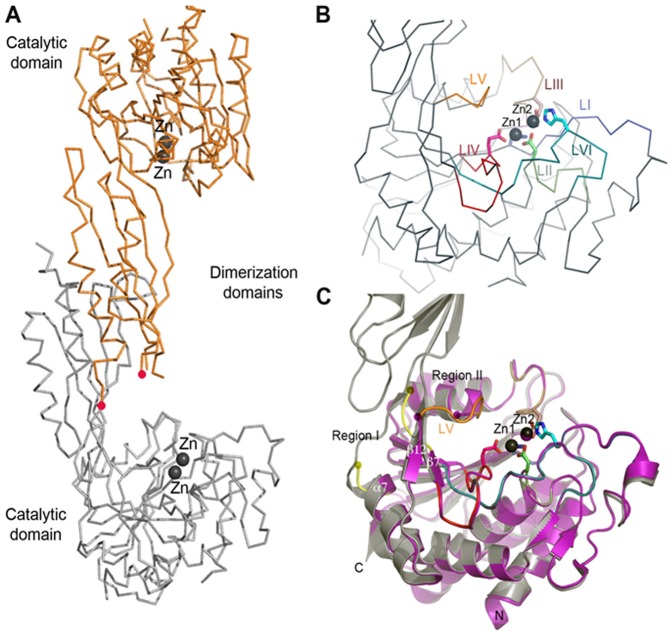
X-ray Crystal Structures of WT-*Hi*DapE and *Hi*DapE^T^. A) Dimer architecture based on the structure of WT-*Hi*DapE. B) Close-up view of the catalytic domain of WT-*Hi*DapE. Ribbon diagram showing the active site formed by six loops (LI–LVI); five of them coordinate the Zn ions (LI–IV & VI). C) Superimposition of the structure of WT-*Hi*DapE (black) over *Hi*DapE^T^ (magenta). Active site Zn(II) ions are shown as black and magenta spheres for WT-*Hi*DapE and *Hi*DapE^T^, respectively. Regions where differences are most prominent are labeled Region I (yellow) and Region II (orange). Yellow and magenta circles highlight two disordered loop regions in the *Hi*DapE^T^ structure. The red dots marked disordered loop that contains conserved His residue.

Deletion of the dimerization domain led to a monomeric structure for *Hi*DapE^T^. The monomeric nature of *Hi*DapE^T^ in solution was corroborated by size exclusion chromatography (SEC) and dynamic light scattering (DLS). The SEC analysis confirmed a weight (MW) of 28.4 kDa (the theoretical mass of a monomer is 28.9 kDa, while the wild-type dimer's theoretical mass is 82.6 kDa) (Supplementary Material Figure S1A in File S1). DLS experiment revealed a monomodal solution containing particles with an average hydrodynamic radius of the 2.52 nm and an estimated average MW of 28.7 kDa (Table 2). The structure of *Hi*DapE^T^ was determined and refined at 1.84 Å resolution and the final model shows two independent monomers in the asymmetric unit. The structure of the catalytic domain is virtually identical to the catalytic domain of full-length *Hi*DapE (PDB ID 3IC1) (Figure 1A, B, & C) [16]. Both structures overlay closely with rmsd for Cα atoms of 0.65 Å over 254 residues. Two zinc atoms were found in the active site and reside in the same position as in dimeric structure of WT-*Hi*DapE separated by 3.40 Å. Superimposition of the active site regions shows nearly identical conformations of main chain and side chains with rmsd as low as 0.15 Å for the metal ions and side chains of residues involved in their coordination (Figure 1C). Therefore the structure of the catalytic core of the enzyme is extremely well preserved in the truncated enzymes. Larger differences within the active site area are observed in two regions containing solvent exposed loops. Region I (residues 176–187) has been engineered to replace what was originally the connector/hinge region formed by two loops linking the catalytic domain with the dimerizaton domain (Figure 1C, Region I). This region is highly flexible and is disordered in the crystal structure of *Hi*DapE^T^ (no electron density observed for residues 182–187). Region II (residues 209–224), which corresponds to a loop overhanging the active site metal ions (loop V, *vide infra*) adopts a different conformation in comparison to that observed in the *Hi*DapE structure (rmsd of 1.5 Å between the structures). Two residues within loop V (Gly21 and Gly212) are disordered in the structure of *Hi*DapE^T^ (Figure 1C).

**Table 2:** Dynamic light scattering data.

Protein	Sample concentration (mg/ml)	*R*_H_ (nm)	MW (kDa)	Pd (nm)	%Pd	Baseline	SOS Error (%)
WT-*Hi*DapE	2	3.94	83.2	0.62	15.8	1.000	0.874
*Hi*DapE^T^	2	2.50	28.7	0.38	15.3	1.000	0.935
*Vc*DapE^T^	2	2.28	23.2	0.52	22.6	1.001	3.19

*R*_H_ – hydrodynamic radius; MW – molecular weight; Pd – the polydispersity, or width of the distribution, in nm, determined using a cumulants analysis (where the data are fit to an assumed distribution of particle sizes and the average radius and spread of radii is reported); %Pd – defined as Pd/*R*_H_, the polydispersity divided by the estimated hydrodynamic radius from the cumulants fit of the autocorrelation function multiplied by 100; Baseline – The measured value of the normalized intensity autocorrelation curve. Values of 1.000 indicate that the measured correlation curve has returned to the baseline within the defined time. Deviations from the theoretical value of 1.000 typically indicate a noisy baseline; SOS error- the sum of squares difference between the measured correlation curve and the best fit curve calculated using the cumulants method of analysis, where a dust and noise free monomodal (single distribution) low polydispersity (narrow distribution) sample is assumed.

### Structure of the catalytic domain of *Vc*DapE^T^

**Figure 2:**
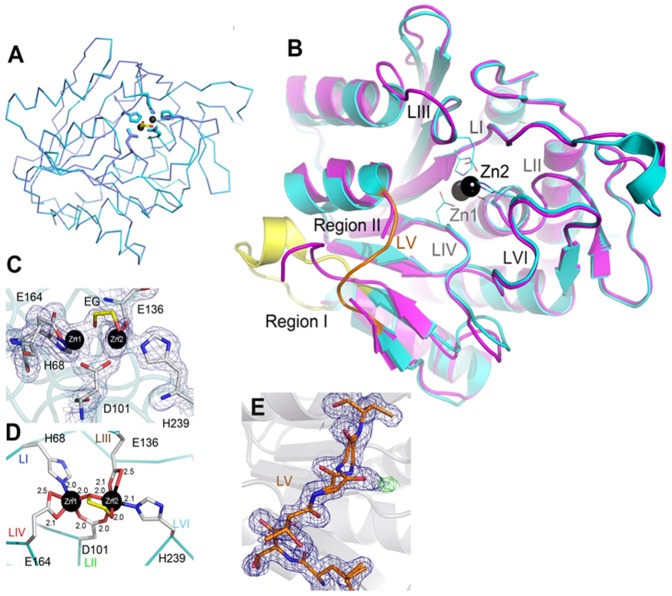
X-ray Crystal Structures of *Vc*DapE^T^. A) Superimposition of apo-*Vc*DapE^T^ (blue) over [ZnZn(*Vc*DapE^T^)] (cyan), showing the identical nature of the catalytic domains. Zinc atoms for *Vc*DapE^T^ are shown as black spheres. B) Comparison of [ZnZn(*Vc*DapE^T^)] (cyan) and [ZnZn(*Hi*DapE^T^)] (magenta). Regions I (yellow) and II (orange) identify the areas where the most significant differences between the two structures exist. Six loops (LI–LVI) forming the active site are labeled. Zinc atoms for *Vc*DapE^T^ are shown as black spheres while the residues coordinating the metal ions are shown as lines. C) Close-up view of the active site environment of the [ZnZn(*Vc*DapE^T^)] with the 2*F*_o_−*F*_c_ electron density map (with the Zn ions and ethylene glycol molecules omitted from the calculation). D) Close-up view of residues from loops I–IV and VI interacting with the Zn(II) ions in the structure of [ZnZn(*Vc*DapE^T^)]. E) The 2*F*_o_−*F*_c_ (blue, 1 σ) and *F*_o_−*F*_c_ electron-density maps (red and green at −3σ and 3σ) of the LV loop region in the apo-*Vc*DapE^T^.

The monomeric state of *Vc*DapE^T^ was confirmed by SEC (the estimated MW of 28.7 kDa) and DLS measurements (the estimated average MW of 23.2 kDa) (Table 2). Two *Vc*DapE^T^ structures were determined, the apo- and the dinuclear Zn(II)-loaded form. The final models for both apo- and Zn(II)-loaded *Vc*DapE^T^ included two molecules of the catalytic domain in the asymmetric unit. The two *V. cholerae* structures are virtually identical and superimpose with the average rmsd of 0.25 Å for 265 equivalent Cα atoms suggesting that binding of Zn(II) does not induce any major structural changes (Figure 2A). The structures of the catalytic domains of both apo- and Zn(II)-loaded *Vc*DapE^T^ closely resemble that of the catalytic domain of active WT-*Hi*DapE (PDB ID 3IC1). Superimposition of the Zn(II)-loaded *Vc*DapE^T^ structure and Zn(II)-loaded *Hi*DapE^T^ shows that they also overlay closely with (the average rmsd of 0.81 Å for 248 equivalent Cα atoms, Z-score 17, sequence similarity 59%) (Figure 2B). Likewise, the structure of Zn(II)-loaded *Vc*DapE^T^ is almost identical to that of the catalytic domain of the full-length *Hi*DapE with rmsd of 1.15 Å. The similarity between these structures is especially apparent within the active site region of Zn(II)-loaded *Vc*DapE^T^ as the active site structure differs by only ∼0.45 Å rmsd with the active site of *Hi*DapE^T^ and ∼0.4 Å with the WT-*Hi*DapE (Figure 2A and B). Similarly to *Hi*DapE^T^, the most flexible regions of the protein are Region I and Region II. Region I corresponds to the engineered loop (the connector) where the deleted dimerization domain is replaced with two glycines. Region II (residues 210–224) forms a loop overhanging the active site metal ions, which includes residues 211–213 that form part of loop V (*vide infra*) (Figure 2B).

### The active sites of *Hi*DapE^T^ and *Vc*DapE^T^

The sequence identity of *Hi*DapE and *Vc*DapE is high (59%), and the residues comprising the active site are fully conserved. The active site of DapE is located in the center of the catalytic domain above the centrally located parallel strands of the β-sheet and is formed by six loops [loop I (*H. influenzae* notation, residues 68–75), loop II (residues 95–102), loop III (residues 132–141), loop IV (residues 162–174), loop V (residues 322–328 in *Hi*DapE) and loop VI (residues 341–355)] (Figure 1B). With the exception of loop V that does not interact with the zinc ions, the remaining loops contribute the conserved residues that coordinate the metal ions (Figures 1B, 1C & 2B). Interestingly, loops I–IV and VI are in virtually identical orientations in both *Vc*DapE^T^ and *Hi*DapE^T^ and overlay very well with the corresponding loops in WT-*Hi*DapE. A change is observed in loop V, which in *Vc*DapE^T^ adopts a conformation that is significantly different from the one observed for WT-*Hi*DapE (Figures 1A & 2B) (in *Hi*DapE^T^ this loop is disordered). The active site of Zn(II)-loaded *Vc*DapE^T^ contains a (μ-oxygen)(μ-carboxylato)dizinc(II) core with one terminal carboxylate and one histidine residue at each metal site ([ZnZn(*Vc*DapE^T^)]), that is identical to the active site observed for WT-*Hi*DapE [16]. The main difference between the active sites in the two Zn(II)-loaded *H. influenzae* structures (WT-*Hi*DapE and *Hi*DapE^T^) and [ZnZn(*Vc*DapE^T^)] is the presence of additional electron density in chain A, which is overlapping with the bridging water/hydroxide molecule observed in chain B and WT-*Hi*DapE. The 2*F_o_-F_c_* and *F_o_-F_c_* electron density maps were quite clear and indicate that a small molecule (most likely ethylene glycol or a partially disordered glycerol molecule) (Figure 2C) displaces the bridging water/hydroxide molecule in the [ZnZn(*Hi*DapE)] and [ZnZn(*Hi*DapE^T^)] structures. Both of these compounds were present in the cryoprotectant solution. Based on the best fit, an ethylene glycol molecule was modeled in the active site resulting in a distorted tetrahedral coordination geometry for Zn1, which is bound by the carboxylate oxygen atoms OD1 of D101 and OE1 of E164 as well as a nitrogen atom NE2 of H68. Zn2 adopts a distorted trigonal bipyramidal geometry and is coordinated by the bridging O2 oxygen atom from ethylene glycol, a nitrogen atom NE2 of H239, two oxygen atoms, OD2 from D101 and OE2 from E136, and the O1 oxygen atom of ethylene glycol (Figure 2D). The Zn1-Zn2 distance is 3.39 Å.

### Molecular Modeling and Dynamics

**Figure 3:**
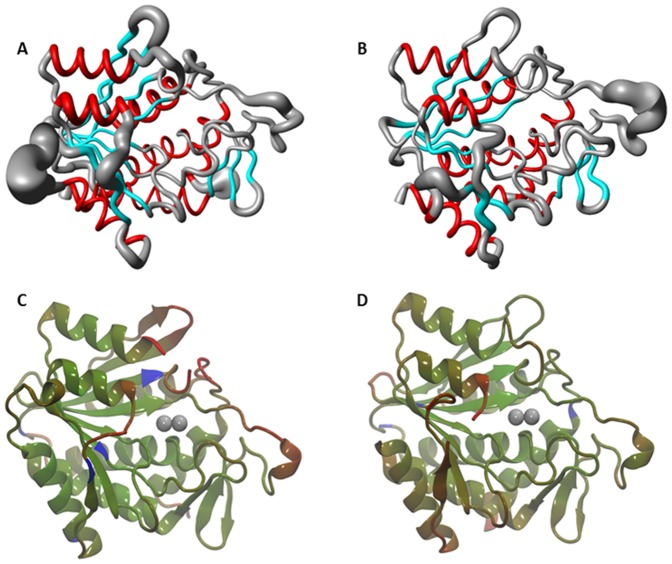
Diagrams Showing Regions of Flexibility in Truncated DapE Proteins. A) MOLMOL diagram of [ZnZn(*Vc*DapE^T^)] molecular dynamics. B) MOLMOL diagram of [ZnZn(*Hi*DapE^T^)] molecular dynamics (the thickness of the line is proportional to the variation of the protein structure during the simulation). The crystallographic temperature factors indicating that the most dynamic (in red) and the most rigid (in blue) parts of the protein: C) [ZnZn(*Vc*DapE^T^)]. D) [ZnZn(*Hi*DapE^T^)].

**Figure 4:**
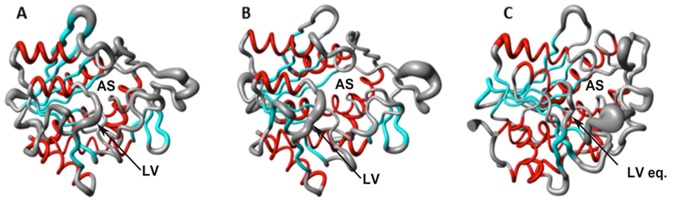
Molecular Dynamic Simulation Showing Regions of Flexibility in Catalytic Domain. A) [ZnZn(*Vc*DapE)]. B) [ZnZn(*Hi*DapE)]. C) AAP. The thickness of the line is proportional to the variation of the protein structure during the simulation. AS indicates the active site area, LVeq. equivalent of the LV loop in *Hi*DapE).

MD calculations were performed for five proteins: *Vc*DapE^T^, *Hi*DapE^T^, WT-*Vc*DapE, WT-*Hi*DapE and AAP. After equilibration, the rmsd values for the main-chain atoms of [ZnZn(*Vc*DapE^T^)] and [ZnZn(*Hi*DapE^T^)] were compared with the original X-ray structures and only small changes were observed. Similarly, small changes were seen after equilibration of *Vc*DapE and *Hi*DapE as well as AAP, which is monomeric and contains a single catalytic domain. Since only the two truncated proteins were catalytically inactive, particular attention was given to how these proteins differed from the other three. Figure 3 shows the results of the 5 ns molecular dynamics simulations of the truncated proteins and comparisons to their crystallographic temperature factors. All of the MOLMOL plots used only the first 5 ns of data so that they are directly comparable. The thickness of the line indicates the flexibility of the structure at a given point during the simulation (Figure 3A and B). The portions of [ZnZn(*Vc*DapE^T^)] that have the greatest flexibility include the created dimerization domain loop and the active site loop V (Figure 2). This is consistent with the crystallographic atomic displacement parameters indicating that the most dynamic portions includes residues 177–187 and loop V (Figure 3C). Compared to the other three structures, the two truncated proteins have greater flexibility in both of these loops (Figure 3) than the wild-type proteins (Figure 4).

### Mutation of the Loop V Residue T325

**Figure 5:**
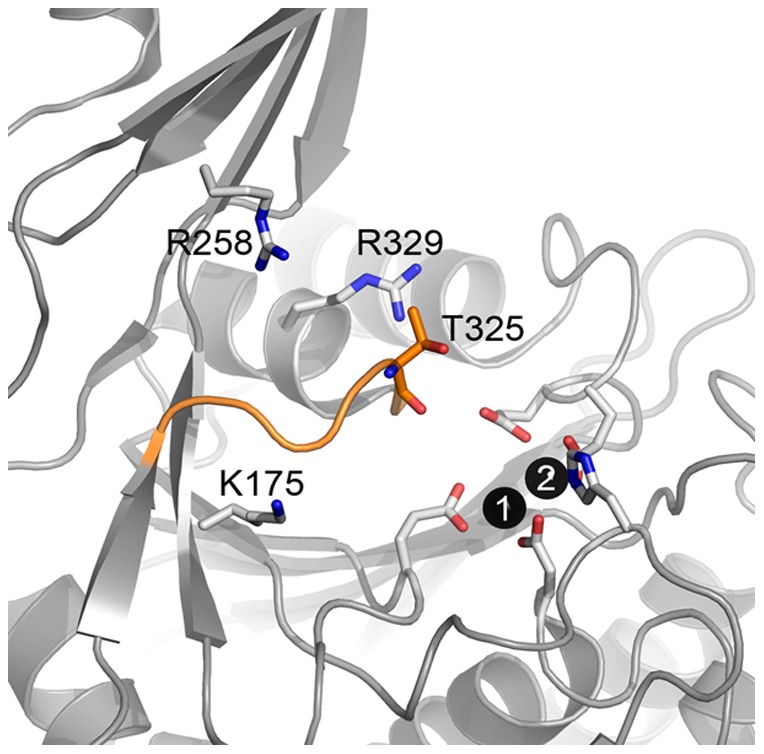
The Active Site of WT-*Hi*DapE Showing Loop V. T325 resides on loop V directly over the dinuclear active site.

Since both X-ray crystallographic and MD simulations suggested that loop V flexibility increases in *Vc*DapE^T^ and *Hi*DapE^T^, it was hypothesized that this loop plays an important role in constituting the active site and possibly substrate recognition and/or transition-state stabilization. Since T325 in *Hi*DapE is centrally positioned in loop V directly above the dinuclear Zn(II) site (Figure 5), the site-directed mutants T325A, T325S and T325C were prepared and purified. The *k*_cat_ value obtained for T325A using L,L-SDAP as the substrate was 4±0.5 s^−1^ with a corresponding *K_m_* value of 2.1±0.2 mM while the *k*_cat_ value obtained for T325S was 2.9±0.3 s^−1^ with a *K_m_* value of 3.0±0.3 mM. Interestingly, when T325 was replaced by cysteine, no enzyme activity was observed under standard assay conditions. CD spectra obtained for each mutant enzyme plus WT-*Hi*DapE, indicate no significant structural change in the mutant enzymes. These data confirm that T325 and hence the position of loop V is important for the active site organization and catalysis.

## Discussion

Sequence comparison of all M20 classified metallopeptidases and the M28 family of dinuclear Zn(II)-dependent metalloproteases, which include AAP and CPG2 [17], [18], [35], shows that all of the metal coordinating residues are strictly conserved [10], [23], [36], [37]. Unlike AAP, which is monomeric, DapE enzymes form dimers where each subunit consists of two functional domains: a larger catalytic domain, which houses the active site and a dimerization domain that forms the dimer interface (Figure 1A). The location and architecture of the catalytic domain and the dinuclear Zn(II) active site in dimeric WT-*Hi*DapE is nearly identical to the catalytic domain and the active site of monomeric AAP [17], [38]. The catalytic domain of *Hi*DapE is composed of residues 1–179 and 293–376 where the core of the catalytic domain contains an eight-stranded twisted β-sheet that is sandwiched between seven α-helices. The active site is located in the center of the catalytic domain above the centrally located parallel strands of the β-sheet and is constituted by six loops. With the exception of loop V that encloses the active site from the top and does not interact with the zinc ions, the remaining loops contribute the conserved residues that coordinate the metal ions (Figure 1B). The dimerization domain of *Hi*DapE consists of a 114-residue insertion into the catalytic domain and is connected to the catalytic domain by two short loops. Since the dimerization domain is located away from the active site and has only limited interaction with the catalytic domain *via* loop V, it is unclear whether this domain plays any role in catalysis. Such a role for the dimerization domain would require a major conformational change to bring the dimerization domain residues into the active site. While this is possible, the time frame would be well beyond the range of atomistic MD simulations.

To address this issue, we engineered DapE enzymes with no dimerization domains (*Hi*DapE^T^, *Vc*DapE^T^). The nested truncation was designed based on the WT-*Hi*DapE crystal structures with the goal of preserving the catalytic domain structure and keep its active site intact. The *Hi*DapE^T^ and *Vc*DapE^T^ constructs expressed well, were purified, the monomeric states of both proteins in solution was confirmed, and their catalytic activities examined. Surprisingly, we could not detect enzyme activity for either truncated variant towards L,L-SDAP, whereas WT-*Hi*DapE exhibited *k*_cat_ and *K_m_* values in good agreement with those previously reported [23]. For comparison purposes, the *k*_cat_ and *K_m_* were also determined for WT-*Vc*DapE and found to be similar to the values reported for WT-*Hi*DapE. These data suggest that the deletion mutants have significantly diminished catalytic activity or are fully catalytically inactive.

To understand the structural basis of the observed absence of catalytic activity of both engineered enzymes, we determined the crystal structures of the truncated forms of *Hi*DapE^T^ ([ZnZn(*Hi*DapE^T^)]) and *Vc*DapE^T^ ([ZnZn(*Vc*DapE^T^)]) along with the apo-form of *Vc*DapE^T^. These structures reveal that the catalytic domain is indeed unchanged, including complete structural preservation of the catalytic site and metal core. It is clear that deletion of the dimerization domain does not compromise the catalytic domain structure. The structure of [ZnZn(*Hi*DapE^T^)] is virtually identical to the catalytic domain the WT-*Hi*DapE with an rmsd of 0.8 Å for 248 equivalent Cα atoms. Interestingly, the newly determined structure of [ZnZn(*Vc*DapE^T^)] also shows high similarity to the catalytic domain of WT-*Hi*DapE with an rmsd of 1.15 Å for 246 equivalent Cα atoms. Since the structures of *Vc*DapE^T^ were determined in both the apo- and dinuclear Zn(II)-loaded forms, it is evident that removal of either the dimerization domain or the metal cations does not alter the overall structure of the catalytic domain.

The dinuclear Zn(II) active sites in both *Hi*DapE^T^ and *Vc*DapE^T^ are nearly identical to each other with Zn-Zn distances of ∼3.40 Å. These Zn-Zn distances are comparable to those observed for AAP (3.45 Å), *Hi*DapE (3.33 Å) and carboxypeptidase G2 (CPG2) (3.25 Å) [18], [37]. Similar to AAP, *Hi*DapE, and CPG2 one of the Zn(II) ions in [ZnZn(*Vc*DapE^T^)] adopts a distorted tetrahedral geometry while the second Zn(II) ion is trigonal bipyramidal. Identical to CPG2, *Hi*DapE, and AAP each zinc ion is coordinated by one imidazole group (H68 for Zn1 and H239 for Zn2) and the oxygen atoms of E164 for Zn1 and E136 for Zn2. Both zinc ions are bridged by D101 and a single oxygen atom of ethylene glycol (in the molecule one, chain A) and a water molecule (in the second molecule, chain B). Overall the [ZnZn(*Vc*DapE^T^)] structure confirms that the metal ions form a (μ-oxygen)(μ-carboxylato)dizinc(II) core similar to *Hi*DapE, AAP, and CPG2 [39], and indicates that the active site catalytic unit is intact in the truncated forms of DapE. Therefore, the lack of the catalytic activity is puzzling and clearly is not due to missing a key element in the *Hi*DapE^T^ and *Vc*DapE^T^ dinuclear active site.

**Figure 6:**
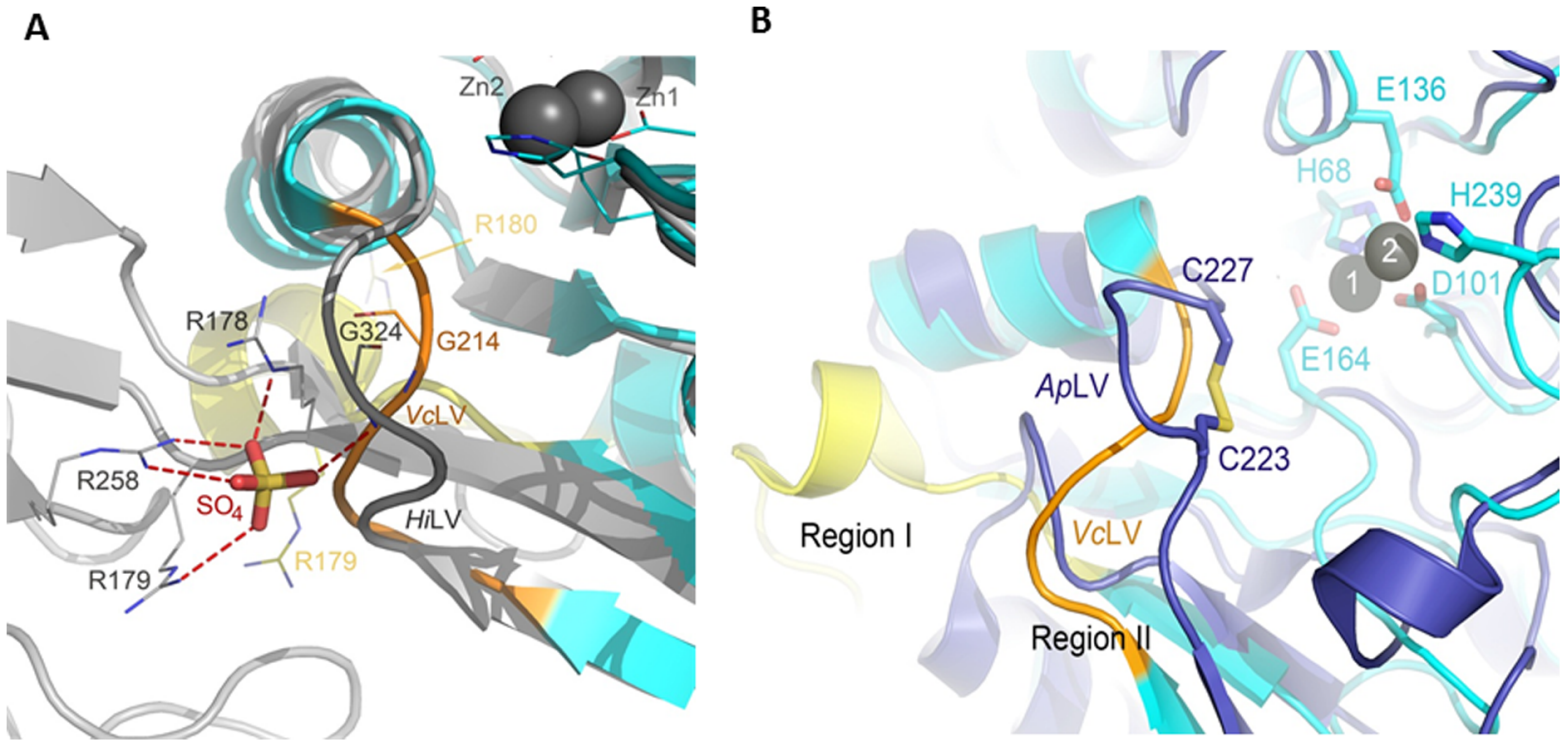
The Role of the Dimerization Domain in the Stabilization of Loop V in WT-*Hi*DapE. A) Superimposition of the WT-*Hi*DapE (gray) and *Vc*DapE^T^ (cyan) structures is shown. Loop V of WT-*Hi*DapE and *Vc*DapE^T^ is labeled as *Hi*LV and *Vc*LV, respectively. WT-*Hi*DapE residues interacting with the sulfate ion (stick model) are shown as gray lines. Corresponding residues in *Vc*DapE^T^ (except for R258 that is absent in the deletion mutant) are shown as yellow (R179 and R180) and orange (G214) lines. B) Specific orientation of the active site loop V in *Vc*DapE^T^ and the corresponding loop in AAP. Overlay of the *Vc*DapE^T^ (cyan) and AAP (purple) structures is shown. The AAP loop and *Vc*DapE^T^ loop V are labeled as *Ap*LV and *Vc*LV, respectively. Stabilization of loop V in AAP by a disulfide bridge is indicated where Cys223 and Cys227 of AAP and the residues involved in zinc-binding in *Vc*DapE^T^ are shown as sticks. Zinc ions of *Vc*DapE^T^ are shown as black spheres. Zinc-bound ethylene glycol was omitted for clarity.

Comparison of the [ZnZn(*Hi*DapE^T^)] and [ZnZn(*Vc*DapE^T^)] structures with that of WT-*Hi*DapE (PDB ID 3IC1) (Figure 1C & 2B) reveals that one of the six loops comprising the catalytic core, loop V (residues 210–214 in *Hi*DapE^T^ and 211–215 in *Vc*DapE^T^, and corresponding to 321–325 in *Hi*DapE) exists in a significantly different orientation than that observed in WT-*Hi*DapE. Interestingly, this loop is also partially disordered in the structure of *Hi*DapE^T^, while in *Vc*DapE^T^ structures its conformational flexibility is signified by the double conformation of the main chain Gly 213 (peptide flip). In previous studies, we proposed that this loop might be involved in the positioning and stabilization of the substrate in the active site, therefore the loop conformation might be critical for proper substrate binding and positioning as well as stabilization of the transition-state during the catalytic cycle [16]. This is supported by two loop V mutations (T325A, T325S) that show similar *K*_m_ to WT but significantly reduced (25–50 times) *k*_cat_ values. These data suggest that the substrate is bound with similar specificity, but is not effectively presented to the catalytic core for successful catalysis. Since the same region is affected in both *Hi*DapE^T^ and *Vc*DapE^T^, where loop V is out of place compared to the WT-*Hi*DapE, we hypothesized that this region is important for catalysis. Both of the observed orientations and the greater flexibility of this loop (Figures 2B & 3) seem to be due to the lack of interactions with specific portions of the dimerization domain. Several interactions may be particularly critical. First, loop V in WT-*Hi*DapE is partially held in position by the anti-parallel beta strands (β7 and β12) and interactions with a loop connecting β7 with the dimerization domain. These contacts are missing in the truncated protein as portions of strands β7 and β12 have been transformed into a deletion-derived loop and α helix (Figure 2B). Loop V in WT-*Hi*DapE is also held in place by the loop from the dimerization domain (residues 223 to 228) (Figure 6). These contacts are also missing in the truncated protein.

Interestingly, the structure of WT-*Hi*DapE contains a sulfate ion located at the interface of the catalytic and dimerization domains and positioned very closely to loop V. The sulfate binding may have biological relevance and might indicate a region where the *L,L*-diaminopimelic acid moiety of the substrates binds. Several residues from both domains interact with this sulfate ion. These include Arg178 (NE), Arg179 (NH), and Gly324 (amide NH; residue in loop V) from the catalytic domain and Arg258 (NH) from the dimerization domain (corresponding to Arg179, Arg180, Gly325 and Arg259 in WT-*Vc*DapE) (Figure 6). This interaction is completely absent in the structure of the [ZnZn(*Vc*DapE^T^)] and residues Arg178 and Arg179 are part of the deletion-derived distorted α helix, whereas Gly214 (corresponding to Gly324 in WT-*Hi*DapE) located within loop V does not form any observable interactions (Figure 6).

Another indication that the specific orientation of loop V may be crucial for catalysis comes from MD simulations of the truncated DapE structures compared to WT-DapE and AAP (Figure 4). Consistent with the large crystallographic temperature factors, the greatest flexibility in the truncated forms of DapE are observed in the engineered loop created to replace the deleted dimerization domain and loop V, which directly overhangs the dinuclear active site (Figure 3C and D). For comparison, loop V in the apo-structure of *Nm*DapE (PDB entry 1vgy) is well ordered and positioned such that it allows two arginine residues (Arg179, Arg259 corresponding to the pairs Arg178/Arg258 and Arg179/Arg259 in *Hi*DapE and *Vc*DapE, respectively) to closely approach the active site. It is highly likely that these Arg residues, which are semi-conserved (Arg178) or conserved (Arg259) in DapEs, might interact with the carboxylate moieties of the substrate. Conserved Arg residues were shown to be essential for substrate binding in human aminoacylase-1 (Acy1) (40). Interestingly in AAP, which exists as a monomeric protein of only the single catalytic domain, there is a loop residing directly over the dinuclear zinc active site that corresponds to loop V in DapE. This loop is very rigid, unlike loop V in DapE, and appears to be held in place by an internal disulfide bridge formed between C223 and C227 (Figure 6). A decrease in dynamic flexibility due to changes in quaternary structure has been suggested for other proteins [40].

Apart from loop V, an additional loop might play an important role in catalysis. Studies on Acy1, another dimeric member of the M20 family, showed that the dimerization domain contributes to the active site through interactions with the substrate (40). Lindner *et al.* (40) showed that the alanine mutant of His206, which is located at the center of the loop, rendered the enzyme nearly inactive. An equivalent histidine residue is conserved in DapE enzymes (H194 in *Hi*DapE and H195 in *Vc*DapE), but it is disordered in the WT-*Hi*DapE structure (17). Modeling of this disordered loop region (Figure 1A) positions H194 roughly ∼13 Å from the active site core, which is likely too far to interact with the substrate. However, it is possible that this loop can rearrange upon substrate binding as was reported for Acy1 and Sapep [40], [41]. Evidence for such loop movement can be gleaned from the structure of apo-*Nm*DapE where this loop is positioned within 8 Å of the active site. These data suggest that this His residue may also play an important role in substrate recognition and binding by DapE enzymes. This hypothesis is consistent with the wild-type DapE structural design as the presence of the inter-domain clefts and connectors would allow for significant movement of this domain. Confirmation of the role of His194 in substrate recognition and binding must await a structure of DapE in complex with a substrate analog.

## Conclusions

Removal of the dimerization domain in DapE enzymes renders the enzyme inactive, even though the resulting truncated proteins have structures of the catalytic domain that are remarkably similar to WT-DapE and AAP. In fact, the dinculear Zn(II) site in the truncated DapE enzymes is nearly identical to those observed for AAP and WT-*Hi*DapE. Therefore, one would expect that the active site should be capable of catalyzing a hydrolytic reaction and yet no activity is detected. This suggests that positioning of the substrate in the active site is critical for the catalytic reaction to occur. Analysis of the structures *Hi*DapE^T^ and *Vc*DapE^T^ reveals that loop V is much more flexible in the truncated enzymes than WT-DapE due to the lack of interactions provided by the dimerization domain. These data suggest that the dimerization domain functions to restrict the conformational freedom of the loop V region and may contribute residues to the active site environment (H194, R179, R258, T325) that are important for substrate recognition, and binding. Designing small molecules inhibitors that interact with this active site loop V region as well as the dinuclear metal center, or disrupting dimer formation may lead to more potent DapE inhibitors that can function as antibacterial agents.

## Supporting Information

File S1 **A.** Analysis of the oligomeric state by size exclusion chromatograpy. Chromatogram showing elution of three DapE proteins (WT-*Hi*DapE (blue), *Hi*DapET (red), *Vc*DapET(green)) from the calibrated column. The inset shows the calibration curve obtained by plotting *K_av_ versus* logMW forthe following standard proteins: Aprotinin (6.5 kDa, I), ribonuclease A (13.7 kDa, II), carbonic anhydrase (29 kDa, III) ovalbumin (43 kDa, IV), conalbumin (75 kDa,V), aldolase (158 kDa, VI), ferritin (440 kDa, VII), and thyroglobulin (669 kDa, VIII). **B.** The secondary structure of WT DapE and T325 mutants were measured using OlisDSM 20 circular dichrometer. Data was collected at room temperature with constant nitrogenflow every 1 nm, in the wavelength range of 190–260 nm. All samples were measured in acylindrical quartz cuvette with a 1 mm pathlength in 10 mM phosphate buffer pH 7.0. Threerepetitive scans were averaged, smoothed and background-subtracted for each measurement. Millidegree values obtained were converted to molar ellipticity (deg·cm2·dmol-1) by using the equation: Molar ellipticity = moM/10×L×C, where mo is millidegrees, M is molecular weight (g/mol), L is path length of cuvette (cm) and C is concentration (g/L).
(DOCX)
