# Supplementary material for: The Dimerization Domain in DapE Enzymes Is required for Catalysis
Source: PLoS One. 2014 May 7;9(5):e93593. doi: 10.1371/journal.pone.0093593 (PMC4012986; doi:10.1371/journal.pone.0093593)
Supplement: File S1 — A. Analysis of the oligomeric state by size exclusion chromatograpy. Chromatogram showing elution of three DapE proteins (WT-HiDapE (blue), HiDapET (red), VcDapET(green)) from the calibrated column. The inset shows the calibration curve obtained by plotting Kav versus logMW forthe following standard proteins: Aprotinin (6.5 kDa, I), ribonuclease A (13.7 kDa, II), carbonic anhydrase (29 kDa, III) ovalbumin (43 kDa, IV), conalbumin (75 kDa,V), aldolase (158 kDa, VI), ferritin (440 kDa, VII), and thyroglobulin (669 kDa, VIII). B. The secondary structure of WT DapE and T325 mutants were measured using OlisDSM 20 circular dichrometer. Data was collected at room temperature with constant nitrogenflow every 1 nm, in the wavelength range of 190–260 nm. All samples were measured in acylindrical quartz cuvette with a 1 mm pathlength in 10 mM phosphate buffer pH 7.0. Threerepetitive scans were averaged, smoothed and background-subtracted for each measurement. Millidegree values obtained were converted to molar ellipticity (deg·cm2·dmol-1) by using the equation: Molar ellipticity = moM/10×L×C, where mo is millidegrees, M is molecular weight (g/mol), L is path length of cuvette (cm) and C is concentration (g/L). (DOCX) [file pone.0093593.s001.docx]

**Supporti****ng Information for**

**The Dimerization Domain in DapE Enzymes is required for Catalysis**

*Boguslaw Nocek^1^, Anna Starus^2^, Magdalena Makowska-Grzyska^1^, Blanca Gutierrez^2^, Stephen Sanchez^2^, Robert Jedrzejczak^3^, Jamey C. Mack^3^, Kenneth W. Olsen^2^, Andrzej Joachimiak^1,3^* and Richard C. Holz^4^**

Contribution from ^1^Center for Structural Genomics of Infectious Diseases, Computation Institute, University of Chicago, 5735 South Ellis Avenue, Chicago, IL 60637, ^2^The Department of Chemistry and Biochemistry, Loyola University-Chicago, 1068 W. Sheridan Rd., Chicago, IL 60626, and ^3^The Midwest Center for Structural Genomics, Bioscience Division, Argonne National Laboratory 9700 S. Cass Ave., Lemont, 60439, and the ^4^Department of Chemistry, Marquette University, Milwaukee, Wisconsin 53201,

**Figure Captions.**

**Figure S1. A.** Analysis of the oligomeric state by size exclusion chromatograpy. Chromatogram showing elution of three DapE proteins (WT-*Hi*DapE (blue), *Hi*DapE^T (^red), *Vc*DapE^T^(green)) from the calibrated column. The inset shows the calibration curve obtained by plotting *K_av_* versus logMW forthe following standard proteins: Aprotinin (6.5 kDa, I), ribonuclease A (13.7 kDa, II), carbonic anhydrase (29 kDa, III) ovalbumin (43 kDa, IV), conalbumin (75 kDa, V), aldolase (158 kDa, VI), ferritin (440 kDa, VII), and thyroglobulin (669 kDa, VIII). **B.** The secondary structure of WT DapE and T325 mutants were measured using Olis DSM 20 circular dichrometer. Data was collected at room temperature with constant nitrogen flow every 1 nm, in the wavelength range of 190 - 260 nm. All samples were measured in a cylindrical quartz cuvette with a 1 mm pathlength in 10 mM phosphate buffer pH 7.0. Three repetitive scans were averaged, smoothed and background-subtracted for each measurement. Millidegree values obtained were converted to molar ellipticity (deg·cm^2^·dmol^-1^) by using the equation: Molar ellipticity = m_o_M/10×L×C, where m_o_ is millidegrees, M is molecular weight (g/mol), L is path length of cuvette (cm) and C is concentration (g/L).

**A**

**
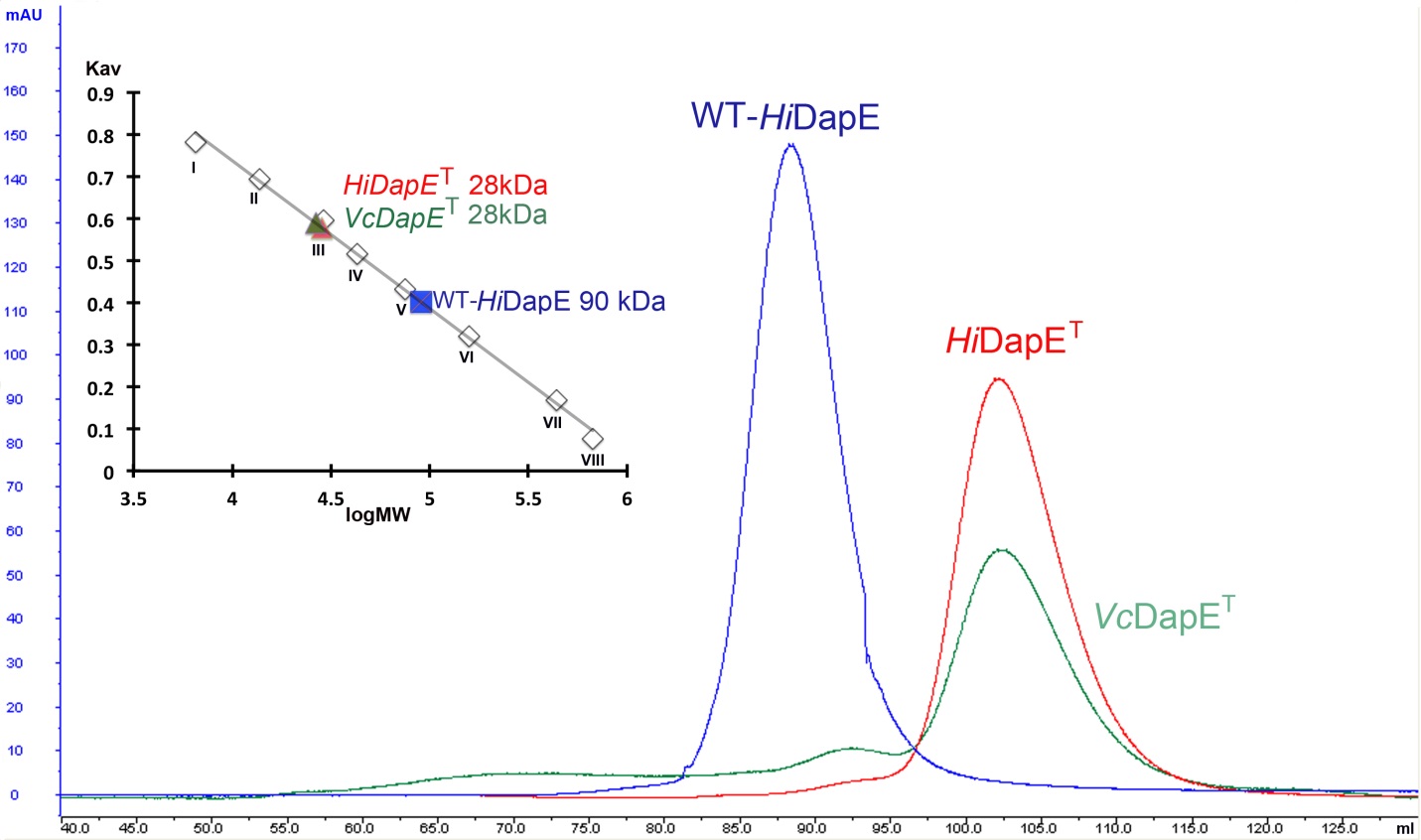
**

**B**

**Table 1.** Analysis of the CD data shown in Figure S2 using the CDSSTR program in Dichroweb.^1^ All proteins are properly folded.

|  | **Helix 1** | **Helix 2** | **Strand 1** | **Strand 2** | **Turns** | **Unordered** | **Total** |
| --- | --- | --- | --- | --- | --- | --- | --- |
| **Wild Type** | 0.24 | 0.05 | 0.20 | 0.12 | 0.17 | 0.23 | 1.01 |
| **T325A** | 0.23 | 0.07 | 0.19 | 0.12 | 0.15 | 0.23 | 0.99 |
| **T325C** | 0.23 | 0.05 | 0.18 | 0.12 | 0.19 | 0.22 | 0.99 |
| **T325S** | 0.30 | 0.06 | 0.17 | 0.11 | 0.14 | 0.21 | 0.99 |

**References**

1. Whitmore, L. W. B. A., DICHROWEB, an online server for protein secondary structure analyses from circular dichroism spectroscopic data. *Nucleic Acids Research* **2004,** *32* (Supplement), 668.
